# Neuroprotective effects of the anticancer drug NVP-BEZ235 (dactolisib) on amyloid-β 1–42 induced neurotoxicity and memory impairment

**DOI:** 10.1038/srep25226

**Published:** 2016-05-04

**Authors:** Paula Maria Quaglio Bellozi, Isabel Vieira de Assis Lima, Juliana Guimarães Dória, Érica Leandro Marciano Vieira, Alline Cristina Campos, Eduardo Candelario-Jalil, Helton José Reis, Antônio Lúcio Teixeira, Fabíola Mara Ribeiro, Antônio Carlos Pinheiro de Oliveira

**Affiliations:** 1 Department of Pharmacology, Universidade Federal de Minas Gerais, Belo Horizonte, 31270-901, Brazil; 2 Department of Biochemistry and Immunology, Universidade Federal de Minas Gerais, Belo Horizonte, 31270-901, Brazil; 3 Department of Internal Medicine, Universidade Federal de Minas Gerais, Belo Horizonte, 30130-100, Brazil; 4 Department of Pharmacology, Universidade de São Paulo, Ribeirão Preto, 14049-900, Brazil; 5 Department of Neuroscience, University of Florida, Gainesville, FL 32610, USA

## Abstract

Alzheimer’s Disease (AD) is a progressive neurodegenerative disease and the main cause of dementia. Substantial evidences indicate that there is over-activation of the PI3K/Akt/mTOR axis in AD. Therefore, the aim of the present study was to investigate the effects of NVP-BEZ235 (BEZ; dactolisib), a dual PI3K/mTOR inhibitor that is under phase I/II clinical trials for the treatment of some types of cancer, in hippocampal neuronal cultures stimulated with amyloid-β (Aβ) 1–42 and in mice injected with Aβ 1–42 in the hippocampus. In cell cultures, BEZ reduced neuronal death induced by Aβ. BEZ, but not rapamycin, a mTOR inhibitor, or LY294002, a PI3K inhibitor that also inhibits mTOR, reduced the memory impairment induced by Aβ. The effect induced by Aβ was also prevented in PI3Kγ^−/−^ mice. Neuronal death and microgliosis induced by Aβ were reduced by BEZ. In addition, the compound increased IL-10 and TNF-α levels in the hippocampus. Finally, BEZ did not change the phosphorylation of Akt and p70s6K, suggesting that the involvement of PI3K and mTOR in the effects induced by BEZ remains controversial. Therefore, BEZ represents a potential strategy to prevent the pathological outcomes induced by Aβ and should be investigated in other models of neurodegenerative conditions.

Alzheimer’s Disease (AD) is the main cause of dementia and affects more than 35 million people^1^,^2^. It is a neurodegenerative progressive disease associated with memory deficits^3^, and its major risk factor is aging^1^. The classical neuropathological markers of AD are extracellular plaques of amyloid-β (Aβ) peptide and neurofibrillary intracellular tangles of hyperphosphorylated tau protein^4^.

The Aβ peptide is originated from the amyloid protein precursor (APP) cleavage by β- and γ-secretase^2^,^4^,^5^. The peptide aggregates and accumulates in the brain as diffuse and compact plaques^6^. Indeed, a wide range of studies shows that intracerebral injection of this peptide in mice can induce AD related cognitive and cerebral changes^7^,^8^,^9^,^10^,^11^,^12^. The disease is characterized by synaptic impairment^7^, neurotrophin and neurotransmitter imbalance, mitochondrial dysfunction, oxidative stress, intracellular calcium increase and cell cycle failure^13^. The most severe changes are in hippocampus, as well as in cortical and subcortical regions^14^, which are associated with the AD memory deficits^15^.

Neuroinflammation is also an important component of the disease, which starts as a defense mechanism against the Aβ deposition in the brain, but can lead to neurodegeneration^9^. The neuroinflammatory process in AD includes disruption of blood-brain barrier and overactivation of glial cells, such as microglia^16^. The microglia role in AD is not completely clear since these cells can have either a beneficial role, phagocyting amyloid plaques, or a deleterious one, releasing inflammatory cytokines and reactive oxygen species^17^.

In spite of all advances in the study of AD, its treatment is still symptomatic, based on cholinergic neurotransmission increase by using acetylcholinesterase inhibitors, e.g. rivastigmine, and reducing glutamatergic hyperexcitability, with memantine^18^. These drugs do not prevent the progression of the disease^19^, being necessary the study of other pathways involved in AD, in order to develop new and more effective pharmacological strategies of treatment.

The regulation of the signaling pathway phosphatidylinositol 3-kinase (PI3K) / protein kinase B (Akt) / mammalian target of rapamicin (mTOR) is important for healthy aging and longevity^1^, since it is involved in cellular metabolism, growing and survival^20^. Abnormalities in this pathway are associated with several conditions, including neurodegenerative processes^1^. The main PI3K target to control cell growth and migration is Akt, which, in turn, phosphorylates various cellular substrates. The activation of Akt leads to the downstream activation of mTOR complex^20^,^21^, which is involved in synaptic regulation and, hence, cognitive processing. The disruption of PI3K pathway can cause detrimental effects on learning and memory processes. In AD, mTOR hyperactivation accounts for abnormal and increased protein translation in synapses^1^,^22^. Studies have shown abnormal and sustained activation of PI3K/ Akt/ mTOR pathway in AD^1^, as well as increased levels of phosphorylated mTOR and decreased cell cycle inhibitors, resulting in mTOR signaling changes^23^. The increased activity of mTOR pathway can induce Aβ production^24^. In addition, the inhibition of PI3Kγ, an isoform of PI3K, by AS605240, following intracerebroventricular Aβ 1–40 injection, reduces the parameters associated with AD, such as astrocyte and microglia cell accumulation in hippocampus, cognitive deficits and synaptic dysfunction^10^. The decreased mTOR signaling can induce the autophagy and lysosomal degradation of Aβ^24^. Thus, dual inhibition of PI3K and mTOR would be a potentially more effective mean to inhibit this pathway^25^,^26^,^27^,^28^,^29^.

Besides, PI3K and mTOR inhibitors are being developed for the treatment of some types of cancer, due to their apoptotic and antiproliferative effects. Importantly, recent studies have demonstrated beneficial effects of anticancer drugs in animal models of Alzheimer’s disease^30^,^31^,^32^,^33^. Therefore, the investigation of an anticancer drug that inhibits PI3K and mTOR may represent a potential therapeutic strategy to treat this pathological condition. NVP-BEZ235 (BEZ; Dactolisib) is an imidazoquinoline, which is in clinical phase studies I/II to treat solid tumors^26^,^34^. It has been demonstrated that this drug can regulate the production of inflammatory mediators in rat primary microglia^35^,^36^. Therefore, in the present study, we evaluated the effects of BEZ on behavioral, biochemical and histological effects induced by Aβ 1–42 *in vitro* and *in vivo*.

## Results

### BEZ prevents neuronal death induced by Aβ in hippocampal neuronal cultures

In order to investigate whether BEZ would prevent the toxic effects of Aβ, we first determined the optimal concentration of Aβ peptide required for inducing neuronal death *in vitro*. The baseline cell death in non-treated neuronal cultures (percentage) was observed in controls (20.760 ± 0.630, n = 3). Incubation of the cultures with Aβ at 0.74 μM, 2.21 μM or 6.64 μM resulted in a significant increase in cell death with percent values of 36.770 ± 2.924 (p < 0.05, n = 4), 33.680 ± 3.339 (p < 0.05, n = 4) and 54.010 ± 5.119 (p < 0.001, n = 3), respectively.

**Figure 1:**
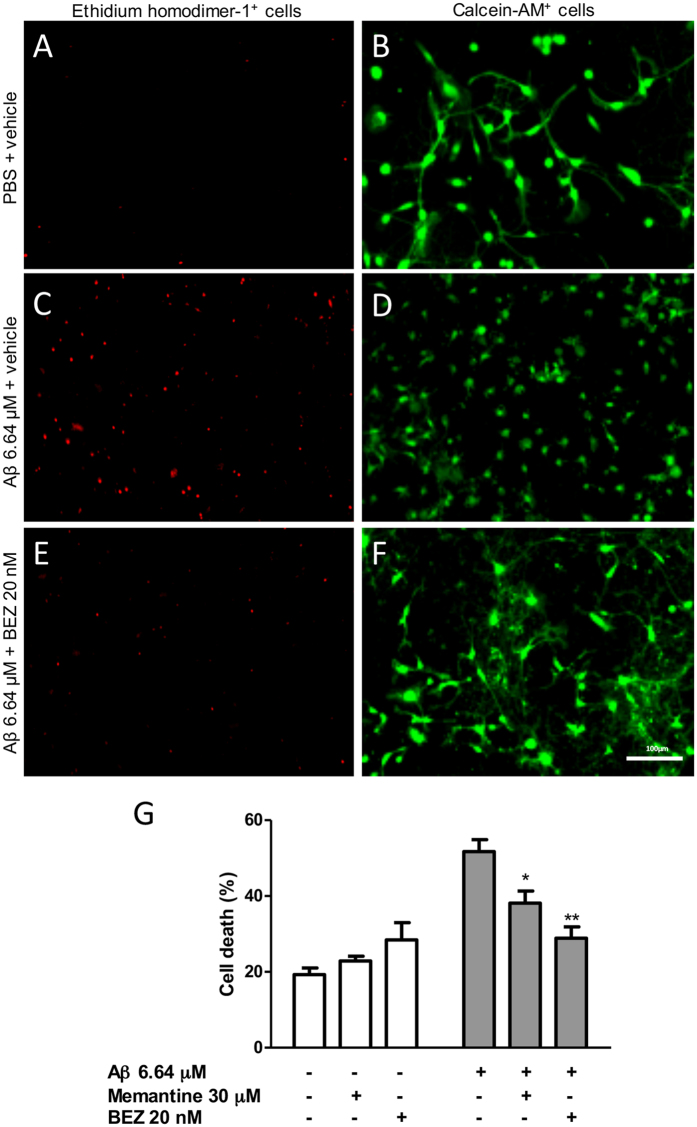
Effect of BEZ on cell death in hippocampal neuronal cultures treated with Aβ. Representative ethidium homodimer-1 and calcein-AM images from treatments PBS + vehicle (**A,B**), Aβ + vehicle (**C,D**) and Aβ + BEZ 20 nM (**E,F**). Bar graphs summarizing the results of the quantification of memantine and BEZ on the cell death induced by Aβ (**G**). Results are expressed as mean ± SEM. *p < 0.05 and **p < 0.01 as compared to cultures treated with Aβ + vehicle (one-way ANOVA followed by Newman-Keuls test).

To evaluate the cell death induced by the drugs themselves, we treated the cultures without Aβ stimulus. There was no significant cell death increase by memantine 30 μM (22.800 ± 1.244, n = 3) and BEZ 20 nM (28.430 ± 4.540, n = 4), when compared with non-treated neuronal cultures (19.260 ± 1.746, n = 8) (Fig. 1A,B). Aβ (6.64 μM) induced a significant increase in cell death (51.710 ± 3.144; p < 0.001, n = 7) (Fig. 1C,D), which was reversed both by memantine 30 μM (38.140 ± 3.166; p < 0.05, n = 4) and BEZ 20 nM (28.900 ± 2.983; p < 0.01, n = 4) (Fig. 1E–G).

### BEZ prevents Aβ induced memory deficits in the Object Recognition Task

**Figure 2:**
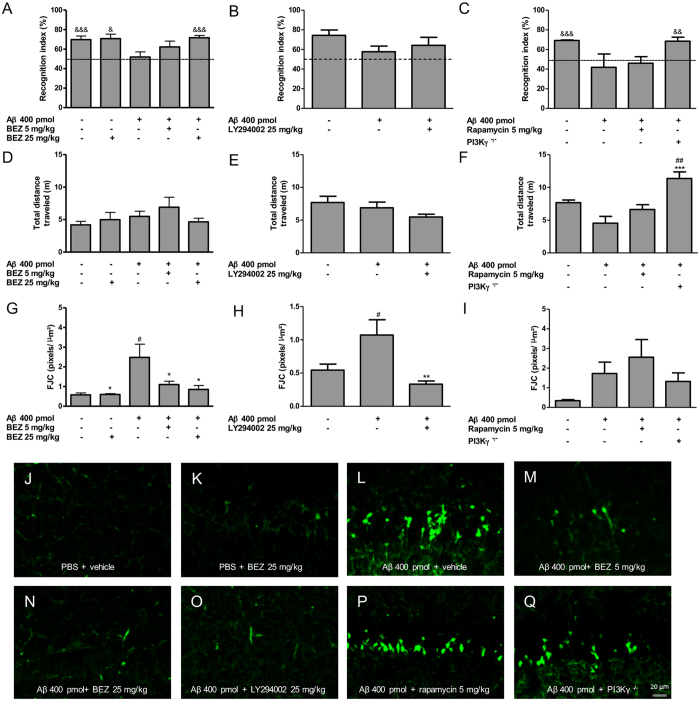
Effect of the treatment with BEZ, LY294002 or rapamycin, and injection of Aβ in PI3Kγ^−/−^ mice on memory deficit and cell death. Plotting results of recognition index, total distance travelled and cell death with treatments with BEZ (**A,D,G**), respectively), LY294002 (**B,E,H**), respectively) or rapamycin (**C,F,I**), respectively), as well as PI3Kγ^−/−^ (**C,F,I**, respectively), 8 days after Aβ injection. Representative FJC slides of ipsilateral hippocampus in 10X magnitude of the groups PBS + vehicle (**J**), PBS + BEZ 25 mg/kg (**K**), Aβ + vehicle (**L**), Aβ + BEZ 5 mg/Kg (**M**) Aβ + BEZ 25 mg/kg (**N**), Aβ + LY294002 25 mg/kg (**O**), Aβ + rapamycin 5 mg/kg (**P**) and Aβ + PI3Kγ^−/−^. Results are expressed as mean ± SEM. ^&^p < 0.05, ^&&^p < 0.01 and ^&&&^p < 0.001 as compared to 50%; *p < 0.05, **p < 0.01 and ***p < 0.001 as compared to Aβ + vehicle; ^#^p < 0.05 and ^##^p < 0.01 as compared to PBS + vehicle (one-way ANOVA followed by Newman-Keuls test).

As we demonstrated a reduction of neuronal cell death induced by BEZ *in vitro*, we further investigated whether this compound could improve the memory deficits induced by Aβ. To assess the memory impairment of mice submitted to the hippocampal Aβ injection, the new object recognition task was performed. Animals that received Aβ and were treated with vehicle presented memory deficits (51.980 ± 5.214%, n = 14), but no memory impairment was observed in groups PBS + vehicle (69.830 ± 3.616%; p < 0.001, n = 14) and PBS + BEZ 25 mg/kg (70.760 ± 4.589%; p < 0.05, n = 4). Moreover, memory deficit induced by Aβ was prevented by the higher dose of BEZ (25 mg/kg; 71.690 ± 2.365%; p < 0.001, n = 11), but not by the lower dose of the compound (5 mg/kg; 62.320 ± 5.901%, n = 8) (Fig. 2A). The total distance travelled did not differ between the groups (Fig. 2D). In order to investigate the involvement of PI3K and mTOR pathway inhibition in the effects mediated by BEZ, we also treated a group of animals, which underwent Aβ injection, with LY294002 (25 mg/kg), a well-known pan PI3K inhibitor that also inhibits mTOR^24^,^37^. We further used another group with rapamycin (5 mg/kg) treatment, a mTOR inhibitor, as well as PI3Kγ knockout (PI3Kγ^−/−^) mice. Memory impairment was not prevented both by LY294002 (64.190 ± 8.174%, n = 7), in spite of the trend to recovery (p = 0.133), and rapamycin (46.070 ± 6.628%; p = 0.57, n = 9). However, PI3Kγ^−/−^ mice did not reveal memory deficit (68.450 ± 4.124; p < 0.01, n = 9) after Aβ injection. The total distance travelled by the animals treated with LY294002 and rapamycin was not statistically different from the other group (Fig. 2B,E), indicating that different recognition indexes of new object over the groups were not related to motor disability. On the other hand, PI3Kγ^−/−^ mice traveled a longer distance (11.360 ± 1.017, n = 9) in comparison with the Aβ + vehicle (4.548 ± 1.030; p < 0.001, n = 4) and PBS + vehicle (7.681 ± 0.404; p < 0.01, n = 5) groups (Fig. 2C,F).

The treatment of the wild-type animals with BEZ, rapamycin or LY294002, as well as the injection of amyloid-β in PI3Kγ^−/−^ mice did not induce body weight (g) changes at any administered doses (data not shown).

### BEZ prevents hippocampal neuronal death induced by Aβ

One mechanism by which BEZ could prevent the memory impairment induced by Aβ would be the reduction of neuronal cell death induced by the peptide. To investigate this issue, we stained hippocampal slices with Fluoro-Jade C (FJC), a gold standard marker for degenerating neurons^38^. Indeed, previously published data demonstrated that FJC staining is increased at 24 h, 8 and 15 days after the injection of the peptide^39^,^40^. Intrahippocampal injection of Aβ induced neuronal death (FJC positive cells; pixels/μm^2^) in the CA1 layer of the ipsilateral hippocampus of animals treated with vehicle (2.481 ± 0.673, n = 11), when compared with groups PBS + vehicle (0.579 ± 0.094; p < 0.05, n = 8) and PBS + BEZ 25 mg/kg (0.598 ± 0.042; p < 0.05, n = 5). Treatment with BEZ 5 mg/kg (1.101 ± 0.168; p < 0.05, n = 7) and 25 mg/kg (0.853 ± 0.201; p < 0.05, n = 8) prevented the Aβ induced neuronal death (Fig. 2G,J–N). There was no significant change in neuronal death in the CA1 layer of contralateral hippocampus among all the groups [PBS + vehicle (0.347 ± 0.065, n = 8), PBS + BEZ 25 mg/kg (0.770 ± 0.146, n = 6), Aβ + vehicle (0.454 ± 0.079, n = 11), Aβ + BEZ 5 mg/kg (0.668 ± 0.140, n = 7) and Aβ + BEZ 25 mg/kg (0.551 ± 0.134, n = 8)].

In addition, we investigated whether PI3K and mTOR inhibition would also reduce FJC staining induced by Aβ.

The treatment with LY294002 reduced the FJC staining in the ipsilateral hippocampus [PBS + vehicle (0.544 ± 0.091, n = 5), Aβ + vehicle (3.315 ± 1.430, n = 4) and Aβ + LY294002 25 mg/kg (0.333 ± 0.048, n = 5); p < 0.05] (Fig. 2H,O), although no changes in FJC staining were found in the contralateral hippocampus [PBS + vehicle (0.381 ± 0.067, n = 6), Aβ + vehicle (0.297 ± 0.031, n = 6) and Aβ + LY294002 25 mg/kg (0.280 ± 0.044, n = 6). No alteration was observed in the neuronal cell death of either wild-type animals treated with rapamycin or in PI3Kγ^−/−^ mice in the ipsilateral [PBS + vehicle (0.346 ± 0.048, n = 6), Aβ + vehicle (1.727 ± 0.579, n = 5), Aβ + rapamycin 5 mg/kg (2.554 ± 0.902, n = 7), Aβ + PI3Kγ^−/−^ (1.322 ± 0.435, n = 5)] (Fig. 2I,P,Q) and contralateral [PBS + vehicle (0.323 ± 0.054, n = 6), Aβ + vehicle (0.576 ± 0.145, n = 5), Aβ + rapamycin 5 mg/kg (0.883 ± 0.364, n = 6), Aβ + PI3Kγ^−/−^ (1.956 ± 0.753, n = 6)] hippocampi.

### Aβ and BEZ do not modify BDNF and NGF levels, as well as caspase-3 activation

Different studies have demonstrated the neuroprotective roles of neurotrophins. Thus, we further investigated whether one mechanism by which BEZ reduces cell death is by increasing the levels of these proteins. However, there was no significant change in BDNF and NGF levels (pg/mg of protein) between groups, both in ipsilateral (BDNF: PBS + vehicle = 187.800 ± 16.730, n = 6; Aβ + vehicle = 193.700 ± 8.438, n = 6; Aβ + BEZ 25 mg/kg = 193.600 ± 28.20, n = 6; NGF: PBS + vehicle = 65.850 ± 6.791, n = 6; Aβ + vehicle = 71.350 ± 2.099, n = 5; Aβ + BEZ 25 mg/kg = 58.96 ± 6.277, n = 6) and contralateral (BDNF: PBS + vehicle = 189.300 ± 11.210, n = 6; Aβ + vehicle = 163.300 ± 11.040, n = 6; Aβ + BEZ 25 mg/kg = 198.800 ± 22.980, n = 6; NGF: PBS + vehicle = 47.350 ± 1.572, n = 6; Aβ + vehicle = 64.210 ± 7.366, n = 5; Aβ + BEZ 25 mg/kg = 57.510 ± 7.116, n = 6) hippocampi.

In addition, in order to investigate whether neuronal death induced by Aβ and its prevention by BEZ is through apoptosis prevention, we determined the levels of caspase-3. Therefore, we determined the ratio between the levels of cleaved caspase-3 and total caspase-3, as well as the ratio between total caspase-3 and actin. Nevertheless, no significant differences were identified between groups PBS + BEZ 25 (107.200 ± 2.719 and 105.600 ± 12.720, n = 4), Aβ + vehicle (99.430 ± 8.458 and 102.500 ± 6.814, n = 5), Aβ + BEZ 5 (103.400 ± 5.698 and 106.500 ± 15.120, n = 5) and Aβ + BEZ 25 (102.900 ± 7.261 and 89.010 ± 5.351, n = 5), for cleaved caspase-3/total caspase-3 and total caspase-3/actin, respectively. The groups were compared to the control group PBS + vehicle, which expression was corrected to 100% for the protein (n = 5).

### BEZ prevents the increased microgliosis induced by Aβ and alters the levels of cytokines in the hippocampus

**Figure 3:**
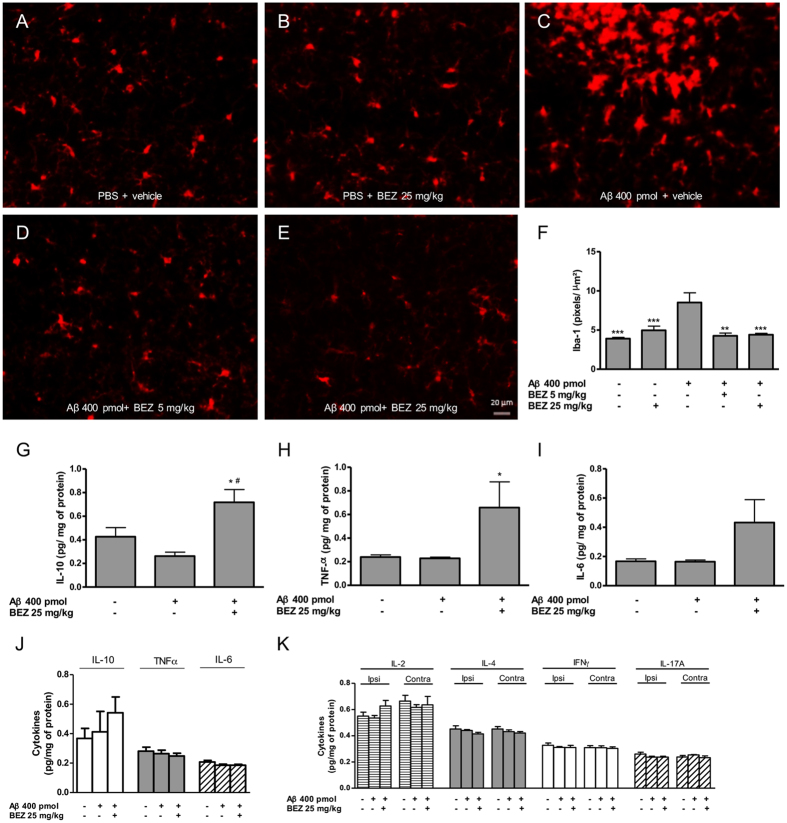
Effect of BEZ on microglia and cytokines expression 8 days after Aβ injection. Representative Iba-1 slides of ipsilateral hippocampus at a 10X magnification in different treatment groups PBS + vehicle (**A**), PBS + BEZ 25 mg/kg (**B**), Aβ + vehicle (**C**), Aβ + BEZ 5 mg/kg (**D**) and Aβ + BEZ 25 mg/kg (**E**). Bar graph summarizing the results of the quantification of Iba-1 data in the ipsilateral hippocampus (**F**). Quantification of IL-10, TNF-α and IL-6 data in the ipsilateral (**G,H,I**), respectively) and the contralateral (**J**) hippocampi. Quantification of IL-2, IL-4, IFN-γ and IL17A in ipsilateral and contralateral hippocampi (**K**). Results are expressed as mean ± SEM. *p < 0.05, **p < 0.01 and ***p < 0.001 as compared to Aβ + vehicle; ^#^p < 0.05 as compared to PBS + vehicle (one-way ANOVA followed by Newman-Keuls test).

As neuroinflammation and microgliosis are important features of AD, we investigated whether the tested doses of the drug were effective to decrease microgliosis through Iba-1 staining, which is a classical marker for microglia^41^. Intrahippocampal injection of Aβ increased microgliosis (Iba-1 positive cells; pixels/ μm^2^) in the CA1 layer of ipsilateral hippocampus of animals treated with vehicle (8.520 ± 1.240, n = 4), when compared with animals in groups PBS + vehicle (3.494 ± 0.155; p < 0.001, n = 4), and PBS + BEZ 25 mg/kg (3.852 ± 0.544; p < 0.001, n = 5). The treatment with BEZ 5 mg/kg (4.264 ± 0.355; p < 0.01, n = 4) and 25 mg/kg (4.402 ± 0.183; p < 0.001, n = 4) prevented the Aβ induced increase in Iba-1 positive cells (Fig. 3A–F). There was no significant change in microglia staining in the CA1 layer of the contralateral hippocampus between groups PBS + vehicle (4.469 ± 0.178, n = 5), PBS + BEZ 25 mg/kg (4.514 ± 0.162, n = 5), Aβ + vehicle (3.845 ± 0.239, n = 4), Aβ + BEZ 5 mg/kg (3.788 ± 0.199, n = 4) and Aβ + BEZ 25 mg/kg (4.624 ± 0.298, n = 4).

Considering that microglia are important sources of cytokines, a reduction in their activation or proliferation could alter the production of these inflammatory mediators. Thus, we investigated the effect of BEZ on the production of various cytokines. We first demonstrated that the levels of IL-10 (pg/ mg of protein) were significantly higher in ipsilateral hippocampus of animals in group Aβ + BEZ 25 mg/kg (0.718 ± 0.108, n = 5) when compared with group PBS + vehicle (0.427 ± 0.077; p < 0.05, n = 5) and Aβ + vehicle (0.262 ± 0.033; p < 0.01, n = 6) (Fig. 3G). There was no significant change in IL-10 levels in contralateral hippocampus (Fig. 3J). TNF-α levels were also significantly increased in ipsilateral hippocampus of animals in the group Aβ + BEZ 25 mg/kg (0.658 ± 0.218, n = 8), compared to groups PBS + vehicle (0.240 ± 0.018, n = 9) and Aβ + vehicle (0.229 ± 0.010, n = 10) (p < 0.05) (Fig. 3H). There was no significant change in TNF-α levels in contralateral hippocampus (Fig. 3J).

Regarding IL-6, one-way ANOVA analysis revealed a general significant difference between all the groups (p < 0.05), albeit post-hoc analysis showed no difference in ipsilateral hippocampus of animals treated with Aβ + BEZ 25 mg/kg (0.433 ± 0.156, n = 8) compared to the groups PBS + vehicle (0.167 ± 0.017, n = 9) and Aβ + vehicle (0.164 ± 0.011, n = 10) (Fig. 3I). There was no significant change in IL-6 levels in contralateral hippocampus (Fig. 3J).

Finally, there was no significant change in the IL-2, IL-4, IFN-γ and IL-17 levels in both ipsilateral and contralateral hippocampi (Fig. 3K).

### BEZ does not change Akt and p70 phosphorylation in the ipsilateral hippocampus

**Figure 4:**
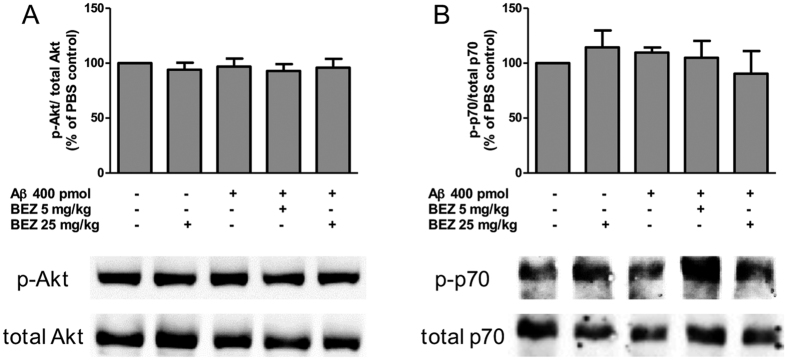
Effect of BEZ on Akt and p70S6K phosphorylation 8 days after Aβ injection. Quantification and representative western blotting images of Akt (**A**) and p70S6K (**B**) phosphorylation in ipsilateral hippocampus in different treatment groups: PBS + vehicle, PBS + BEZ 25 mg/kg, Aβ + vehicle, Aβ + BEZ 5 mg/kg and Aβ + BEZ 25 mg/kg. Results are expressed as mean ± SEM.

We next investigated whether BEZ would reduce the activation of Akt and p70s6 K, two indirect methods to determine PI3K and mTOR activities, respectively, in the hippocampus. The injection of Aβ did not induce significant changes in Akt and p70S6 kinase phosphorylation (96.880 ± 7.349%, n = 4 and 109.700 ± 4.686%, n = 3, respectively) when compared to the control group PBS + vehicle, to which the expression was normalized to 100%. The phosphorylation of Akt and p70s6K was also not modified in animals treated with BEZ (25 mg/kg) that received PBS (94.050 ± 6.424%, n = 3 and 114.400 ± 15.36%, n = 4, respectively), as well as in animals that received BEZ (5 mg/kg) (92.920 ± 6.254%, n = 4 and 104.900 ± 15.360%, n = 4, respectively) or BEZ (25 mg/kg) (95.930 ± 8.062%, n = 4 and 90.450 ± 20.66%, n = 4, respectively) that were treated with Aβ (Fig. 4A,B).

## Discussion

In the present study, we demonstrated that Aβ caused neuronal death both *in vitro* and *in vivo*, as well as memory impairment and increased microgliosis in hippocampus of mice. We showed that the treatment with BEZ, an anticancer drug, prevented all these pathological alterations. In addition, BEZ increased the levels of IL-10 and TNF-α and there was a trend toward an increase in IL-6 levels in the hippocampus following injection with Aβ.

After the diagnosis of AD, the disease generally leads patients to cognitive impairments and death into about 3 to 9 years^13^. Memory impairment and other cognitive deficits are associated with increased dependence and incapacity in AD^42^. These symptoms are related to the functions of cerebral structures affected, specially hippocampus and neocortical areas^15^. Once there is no effective therapy against AD progression, there is a great need of pharmacological development in this area^43^.

The PI3K/Akt/mTOR pathway plays an important role in the integration of synaptic signaling^20^. A series of evidences suggest that the increase in cell cycle events, loss of neuronal processes and neurotoxicity after exposition to Aβ depends on the activation of PI3K pathway. A previous study showed that the blockade of PI3K with wortmannin in mixed neuron-glia cultures treated with Aβ reduces microglia activation^44^. Therefore, the inhibition of this pathway might represent a potential therapeutic strategy for the treatment of AD^45^. The inhibition with a drug targeting both PI3K and mTOR would be useful, once it would avoid retrograde activation of Akt usually observed after treatment with mTOR inhibitors^26^.

According to previous reports, Aβ injection led to memory impairment^10^,^39^,^40^,^46^, which was prevented by the BEZ treatment. However, whether PI3K and mTOR inhibition mediates the effects on memory induced BEZ is still controversial. First, we demonstrated that LY294002, a compound known to inhibit PI3K and mTOR, as well as rapamycin, which inhibits mTOR, did not avoid memory impairment in this context. Second, BEZ also did not alter the phosphorylation of Akt and p70s6K in the hippocampus 4 h after the treatment. On the other hand, the PI3Kγ^−/−^ mice were resistant to the memory impairment induced by Aβ, a result that corroborates a previous study which showed that a sole PI3Kγ inhibition (with AS605240) prevented learning deficits induced by Aβ 1–40 in the Morris Water Maze^10^. Nevertheless, further studies would be necessary to investigate whether the inhibition of PI3Kγ is responsible for the beneficial effects of BEZ in this model. Besides, other currently unknown mechanisms may also be involved.

We also demonstrated that BEZ reduced *in vitro* and *in vivo* neuronal cell death induced by Aβ. Interestingly, LY294002 also reduced FJC staining induced by Aβ, albeit it did not prevent memory impairment. Indeed, different studies demonstrated that LY294002 reduced memory improvement induced by other drugs, albeit it has no effect on memory *per se*^47^,^48^. Thus, even though a reduction in neuronal cell death induced by LY294002 would improve memory deficits, this effect would be counteracted by its direct effect on cognition. On the other hand, PI3Kγ^−/−^ mice were resistant to the memory impairment, but not neuronal death, induced by Aβ. Importantly, the neuronal cell death observed in the present model is not as intense as observed in other models, such as in neurodegeneration induced by excitotoxic stimuli. Thus, considering that there is a dichotomy between neuronal death induced by Aβ injection and memory, it is possible that different mechanisms underlie the beneficial effects of BEZ.

Since BDNF and NGF promote proliferation, differentiation and survival of neurons and glial cells, as well as mediate cognitive and behavioral responses, we measured their levels in the hippocampus. Other studies have shown a decrease in neurotrophin receptors in cholinergic neurons of patients with AD^13^. It has also been shown that BDNF is reduced both in brains of patients with AD and in cell cultures treated with Aβ, what appears to be dependent on the age and pathology progression^49^. Moreover, treatment with either BDNF or NGF in animal models of AD is able to improve some features associated with the disease, like the memory impairment^13^. Notwithstanding, in our model no difference in the levels of these neurotrophins were detected 7 days after the peptide infusion or the drug treatment. This discrepancy might be related to the disease models used in different studies and to the different periods between the peptide infusion and the determination of the neurotrophins levels. In addition to this, we observed that cleaved and total caspase-3 levels were not changed by BEZ, suggesting that the neuronal cell death induced by Aβ and the effect of BEZ might not be related to reduction of this molecule.

Another mechanism related to AD is neuroinflammation. Immune cells, such as microglia, migrate and accumulate in the vicinity of Aβ plaques, leading to plaque phagocytosis and Aβ degradation^16^,^17^,^50^. However, due to a sustained activation, microglia also releases pro-inflammatory cytokines, neurotoxins and other substances that can lead to neuronal death^9^,^51^. The chronic inflammatory process, associated with the production of inflammatory mediators and cellular stress, increases APP amyloidogenic processing, causing a vicious cycle^52^. As in a previous study^50^, we demonstrated here that Aβ increased microgliosis, which was prevented by BEZ treatment. This effect could contribute to the reduced neurotoxicity, and could be associated with the cognitive improvement after the drug treatment.

Different studies have shown that cytokines may be involved in the pathogenesis of AD and cognitive dysfunction. IL-10 is a neuroprotective cytokine that interacts with cell surface receptors, especially in glia^52^. IL-10 has some behavioral effects arising from pro-inflammatory cytokines inhibition, showing its potential to ameliorate neuroinflammation, cognitive deficits and neurodegeneration. Previous data showed that this cytokine is able to reduce microgliosis, improve spatial learning in the radial arm water maze and enhance neurogenesis in a transgenic model of AD^53^. Therefore, the improvement in Object Recognition Task could be related to IL-10 increased levels after treatment with BEZ.

TNF-α plays a central role in cytokine production cascade during the inflammatory response, being predominantly produced by microglia in AD^52^. Several authors have demonstrated that increased expression of TNF-α participate in the neuroinflammation associated with AD^54^,^55^,^56^. On the other hand, it has been demonstrated a neuroprotective role of this cytokine against glutamate, free radicals and Aβ induced toxicity in cultured neurons^52^,^57^. In AD, IL-6 levels are also altered, with increased expression in the vicinity of Aβ plaques and in the cerebrospinal fluid of patients. In spite of stimulating the synthesis of APP in glial cell cultures, increasing the damage in cortical neurons cultures stimulated with Aβ^58^, IL-6 may also have beneficial roles. Studies with transgenic models for AD showed that IL-6 was important to promote gliosis, leading to clearance of amyloid plaques^58^,^59^,^60^. Herein, BEZ increased TNF-α and showed a trend to increase in IL-6 levels in the ipsilateral hippocampus. Is has been previously shown that BEZ enhances the levels of other inflammatory mediators, such as COX-2, and prostaglandins, such as PGE_2_ and PGD_2_ in LPS-stimulated microglia^35^,^36^. Nevertheless, no difference in the levels of these cytokines was detected 7 days after the peptide infusion. This could be related to variations between the model and the protocol adopted by our study in comparison with the others.

In this study, we demonstrated that the treatment with BEZ, a PI3K and mTOR inhibitor with anticancer properties, improves mice performance in the object recognition task after intrahippocampal Aβ administration. In parallel with this cognitive effect, treatment induces neuroprotective effects, preventing cell death and reducing microgliosis. These effects might be related to the change in the production of different cytokines, albeit the mechanism remains unclear. Thus, BEZ might represent a potential drug to prevent the pathological outcomes induced by Aβ. However, more studies are necessary in order to investigate the protective mechanism promoted by BEZ in transgenic AD models.

## Methods

### Culture of primary hippocampal neurons

Neuronal cultures were prepared from the hippocampus of C57Bl/6 mice neonates up to 1 day of age. After dissection, hippocampal tissue was subjected to digestion with trypsin, followed by cell dissociation. Cells were added to the Neurobasal medium supplemented with N2 and B27, GlutaMAX (2.0 mM), penicillin (50.0 μg/ml) and streptomycin (50.0 mg/ml), and then plated on previously prepared poly-L-ornithine four well plates. The cells were incubated at 37 °C and 5% CO_2_ in a humidified incubator and cultured for 8 days, with medium change every 4 days. Importantly, we have previously established that 95% of the cells in these cultures are neurons^61^.

### Cell death assay

Cell death assay was performed using the Live/ Dead kit (Life Technologies). Neurons were incubated for 20 h in the presence of PBS or Aβ 1–42 (6.64 μM), and treated with DMSO, memantine (30 μM) or BEZ (20 nM). After incubation, neurons were labeled with calcein-AM (2.0 μM) and ethidium homodimer-1 (2.0 μM) solution for 15 minutes in the incubator. Afterward, neurons were washed 3 times with PBS.

Photographs were taken with the microscope EVOS® FLoid® Cell Imaging Station, using 488 nm filter for green images (Calcein-AM) and 633 nm for red images (ethidium homodimer-1). Images were analyzed with ImageJ software. The number of dead cells was expressed as a percentage of total cell number.

### Animals

All procedures used in this study were approved and strictly followed the ethical principles of animal experimentation adopted by the Ethic Committee on Animal Use of Federal University of Minas Gerais, and institutionally approved under protocol number 336/2012. Experiments were conducted using male C57Bl/6 mice (25–30 g, 10–12 weeks of age) obtained from Animal Care Facilities of the Institute of Biological Sciences (ICB), and PI3Kγ^−/−^, which were a kind gift from Prof. Mauro M. Teixeira, from ICB - UFMG, Belo Horizonte, Brazil. Animals were kept under controlled room temperature (24 °C) under 12 h:12 h light-dark cycle, with free access to food and water. In total, 86 animals were used in this study.

### Drug treatment protocol

Human Aβ 1–42 (Invitrogen) was prepared according to manufacturer instructions. The aggregated peptide (400 pmol/ 0.5 μL/mice) or PBS (vehicle) was administered via intra-hippocampal route. Briefly, the animals were anesthetized with an intraperitoneal (i.p.) injection of ketamine (80 mg/kg) and xylazine (8 mg/kg) and then submitted to stereotactic surgery and intrahippocampal injection. The needle was inserted unilaterally and Aβ 1–42 or PBS solution was injected into the right hippocampus at the following coordinates from bregma^62^: anteroposterior = −1.9 mm, mediolateral = −1.5 mm, and dorsoventral = −2.3 mm. The confirmation of the correct placement of the needle was made using Cresyl Violet staining (data not shown).

Animals were treated by oral gavage with de dual PI3K and mTOR inhibitor BEZ (2-Methyl-2-(4-[3-methyl-2-oxo-8-(quinolin-3-yl)-2,3-dihydro-1 H-imidazo[4,5-c]quinolin-1-yl]phenyl)propanenitrile) (LC Laboratories) (5 or 25 mg/kg), diluted in 1-metyl 2-pirrolidone 10% in polyethylene glycol 300, 1 h prior to Aβ 1–42 injection and once a day for 7 days. According to the intra-hippocampal injection (PBS or Aβ 1–42) and the treatment (BEZ or vehicle), they were divided into 5 groups: PBS + Vehicle, PBS + BEZ 25 mg/kg, Aβ + vehicle, Aβ + BEZ 5 mg/kg and Aβ + BEZ 25 mg/kg. The doses of BEZ were chosen based on previous published data^63^,^64^,^65^,^66^. Animals were weighed every day before the drug administration. Other animals were treated intraperitoneally with LY294002 (LC Laboratories) 25 mg/kg diluted in DMSO 5%, ethanol 5%, polyethylene glycol 400 5% in sodium chloride 0.9%; or rapamycin (LC Laboratories) 5 mg/kg diluted in ethanol 4%, polyethylene glycol 400 5%, tween 80 5% in sodium chloride 0.9%. The treatment was 1 h prior to Aβ 1–42 injection and once a day for 7 days. In addition, Aβ 1–42 was microinjected also in PI3Kγ^−/−^ mice. For the investigation of the effect of LY294002 on memory, the following groups were used: PBS + vehicle, Aβ + vehicle, Aβ + LY294002 25 mg/kg. To evaluate the effect of rapamycin and genetic deletion of PI3Kγ, the following groups were used: PBS + vehicle, Aβ + vehicle, Aβ + rapamycin 5 mg/kg, Aβ in PI3Kγ^−/−^ mice. The doses of LY294002 and rapamycin were chosen based on previous published data^67^,^68^,^69^,^70^,^71^.

### Object Recognition Task

On the 4^th^ day after surgery, the Object Recognition Task was started. The animals were habituated during 5 minutes in an acrylic square box, dimensions 380 × 380 × 15 mm (length × width × height), covered with shavings. On the 5^th^ and 6^th^ days the animals were re-exposed to the box, in which 2 equal objects were introduced diagonally. Animals were let 10 and 5 minutes inside de box, in the 2 subsequently days, respectively. On the 7^th^ day, one of the old objects (OO) was replaced by a new object (NO), and the animals were exposed for 5 minutes^72^,^73^. The records were analyzed through ANY-maze software version 4.99, and the recognition index was obtained by the formulae: time NO × 100/ (time NO + time OO). The total travelled distance was also measured as a control of the test. Animals that did not investigated the objects were not included in the analysis.

### Intracardiac perfusion and brain slice preparation

In the last day of treatment, 4 hours after the drug administration a subgroup of animals were anesthetized with ketamine (80 mg/kg) and xylazine (8 mg/kg) via i.p. route and then were subjected to thoracotomy to expose the heart. A hypodermic needle was inserted into the left ventricle, through which PBS and buffered paraformaldehyde (PFA) 4% were administered with the assistance of a peristaltic pump (4 mL/min). Meanwhile, an incision was made in the aortic arch to allow blood output. After completing the perfusion, the animals were decapitated, their brains were removed and stored in buffered PFA 4% overnight. Subsequently, the brains were moved to a 30% sucrose solution, until complete saturation, then were frozen in isopentane 99% and dry ice for 20 seconds and stored at −80 °C^74^. Brains were sliced into 30-μm-thick sections at −20 °C with the aid of a cryostat.

### Fluoro-Jade C staining

Fluoro-Jade C (FJC) is an anionic fluorescein derivate used to label degenerating neurons^38^. Although the exact mechanism is not known, different studies have demonstrated that FJC is a reliable dye used to stain dying neurons^75^,^76^,^77^,^78^,^79^. The hippocampal slices were washed 3 times in PBS for 30 minutes and mounted on gelatinized slides. After drying, slides were immersed in a basic solution of sodium hydroxide (1%) in ethanol (80%) for 5 min, EtOH (70%) for 2 minutes and rinsed with distilled water for 2 minutes. Protected from light, the slides were incubated in a solution of potassium permanganate (0.06%) for 20 minutes, washed with distilled water for 2 minutes and incubated in FJC (Millipore, Billerica, MA, USA) solution (0.0001%) in acetic acid (0.1%) for 20 minutes. Subsequently, they were washed again twice with distilled water for 1 min. After complete drying, slides were dipped in xylene for 1 minute and coverslipped with DPX (Sigma-Aldrich, St. Louis, MO, USA)^78^.

The slides were observed under fluorescence microscope Zeiss in 10× magnification lens and pictures of the CA1 layer of both hippocampi were taken for quantification of labeled cells.

### Iba-1 staining

The hippocampal slices were washed 3 times in Tris-Buffered Saline (TBS) for 30 minutes. Free-floating slices were incubated with citrate buffer at 70 °C for 30 minutes, for antigen retrieval. After, blocking solution [BSA (4%), Triton X (0.5%) in TBS] was added to the slices for 2 h, and they were incubated overnight with anti-Iba-1 primary antibody (1:500; Wako Chemicals, Osaka, Japan). On the next day, the slices were incubated with the secondary antibody Alexa Fluor 594 anti-rabbit (1:1000; Invitrogen, Carlsbad, CA, USA) for 1 h, washed, mounted in gelatinized slides and coverslipped with Fluoromount media (Sigma-Aldrich, St. Louis, MO, USA)^77^.

The slides were observed under a Zeiss fluorescence microscope in 10× magnification lens. Pictures of the CA1 layer of both hippocampi were taken for quantification of labeled cells.

### Analyses of neurotrophins and cytokines

Animals that did not underwent intracardiac perfusion were anesthetized with ketamine (80 mg/kg) and xylazine (8 mg/kg) i.p., and had both their right and left hippocampus dissected after the behavior studies. For biochemical analyses, the tissue was homogenized in 200 μL of a buffer containing protease inhibitors [NaCl (0.4 M); Tween 20 (0.05%); Bovine Serum Albumin (BSA) (0.5%); phenylmethylsulfonyl fluoride (PMSF) (0.1 mM); benzethonium chloride (0.1 mM); EDTA (10 mM); aprotinin (20 IU) in PBS]. Total proteins were measured by Bradford method^80^ and analyzed by Enzyme Linked Immunosorbent Assay (ELISA) to measure BDNF and NGF (kits DuoSet® R&D Systems), or Cytometric Bead Array (CBA), (kit Th1/Th2 BD) to detect IL-2, IL-4, IL-6, IFN-γ, TNF-α, IL-10 and IL-17 A. All the procedures followed manufacturer’s instructions.

### Evaluation of Akt, p70 and caspase-3 by western blotting

In the last day of the treatment, hippocampal tissues of the animals were carefully dissected 4 h after the drug administration, homogenized in a lysis buffer (1% Triton X-100; 100 mM Tris/HCl, pH 8.0; 10% glycerol; 5 mM EDTA; 200 mM NaCl; 1 mM DTT; 1 mM PMSF; 25 mM NaF; 2.5 μg/ml leupeptin; 5 μg/ml aprotinin; and 1 mM sodium orthovanadate) and stored in −80 °C until the beginning of the analysis. Protein concentration was determined by using the Bradford protein assay (Bio-Rad, Hercules, CA, USA). Fifty μg of protein samples were separated on 10% SDS-polyacrylamide gels and then transferred to nitrocellulose membranes. After blocking in 10% bovine serum albumin (BSA) in Tris-buffered saline containing 0.1% Tween-20 (TBST) for 2 h at room temperature, membranes were incubated overnight at 4 °C with primary antibodies against anti-phospho-AktSer473 (1:1000; DB Biotech), anti-Akt1 clone C20-A (1:1000; DB Biotech), anti-phospho-p70 S6 Kinase (T389) (1:250; Cell Signaling), anti-p70 S6 Kinase (1:250; Cell Signaling), anti-caspase-3 (1:1000; Cell Signaling) and anti-Actin (20–33) (1:5000; Sigma- Aldrich). Following three washes with TBST, membranes were incubated with the appropriate peroxidase-conjugated secondary antibodies (1:2500). Finally, membranes were incubated with enhanced chemiluminescence ECL-Plus (GE Healthcare). The optical densities of detected bands were quantified using the ImageJ software. The results were normalized to the levels of β-actin in each sample lane.

### Statistical Analysis

Statistical analysis was performed using the statistical software GraphPad Prism 5.0. Body weight data was analyzed by two-way analysis of variance (ANOVA) followed by Bonferroni’s test for variables with parametric distribution. Behavioral, biochemical, histological and *in vitro* data was analyzed by one-way ANOVA followed by Newman-Keuls test for variables with parametric distribution. The data were presented as mean ± standard error of the mean (SEM). The level of significance was set at p < 0.05.

## Additional Information

**How to cite this article**: Bellozi, P. M. Q. *et al*. Neuroprotective effects of the anticancer drug NVP-BEZ235 (dactolisib) on amyloid-β 1–42 induced neurotoxicity and memory impairment. *Sci. Rep*. **6**, 25226; doi: 10.1038/srep25226 (2016).
